# Unlocking the potential of genetic resources in the Pangenome era

**DOI:** 10.1270/jsbbs.75.1

**Published:** 2025-03

**Authors:** Sachiko Isobe

**Affiliations:** 1 Graduate School of Agricultural and Life Sciences, The University of Tokyo

The rapid advancements in next-generation sequencing (NGS) technologies have transitioned genome analysis from the era of single-reference genomes to the Pangenome era, where the genomes of multiple individuals and lineages are analyzed in an integrated manner. This transformation has unveiled a range of genomic variations that were previously overlooked in single-reference genome analyses, including chromosomal structural variations such as translocations and deletions, as well as structural variants. Additionally, Pangenome analyses have identified presence-absence variations (PAVs) and novel genetic factors that were previously unknown (Bayer *et al.* 2020, Golicz *et al.* 2016). These insights provide unprecedented opportunities for utilizing genetic diversity in breeding programs.

At the same time, agriculture is facing severe challenges due to climate change and the increasing global demand for food. Crop improvement strategies must not only aim to enhance yield but also focus on developing varieties with disease resistance and environmental adaptability to maintain stable, with high productivity under diverse conditions. To achieve this, it is essential to effectively utilize the diverse genetic traits found in crop genetic resources and wild relatives (Zhao *et al.* 2018). By integrating Pangenome analysis into breeding science, the discovery of beneficial alleles, structural variations, and adaptive traits can be accelerated, facilitating the development of crops that are more resilient to climate change (Tao *et al.* 2019).

This special issue aims to explore the impact of Pangenome research on breeding and the utilization of genetic resources. It includes six review articles and one research paper that showcase examples of how Pangenome analysis can enhance the breeding of a wide range of genetic resources. Since advances in NGS technologies are essential for Pangenome analysis, the first paper by Aoyagi Blue *et al.* (2025) discusses the importance of telomere-to-telomere genome assemblies in the plant Pangenome era while touching upon the evolution of NGS technologies. Next, as an example of Pangenome analysis in diverse crop genetic resources, Sato (2025) provides insights into the genetic diversity of barley and the latest findings from Pangenome analysis. Following this, Shimizu and Nonaka (2025) explore how genomics unlock the potential of genetic resources in citrus breeding through Pangenome analysis of diverse genetic resources. Furthermore, Kato (2025) reviews the current status and future perspectives of Pangenome research in cucurbit crops. As an approach that actively utilizes wild genetic resources, Yoshikawa and Sato (2025) discuss the application of Pangenome analysis in the breeding of wild Oryza species. Additionally, as a practical research example, Chapman (2025) reports on novel breeding resources for the underutilized legume Lablab based on a Pangenome approach. Finally, as an epigenetic analysis approach, Lam *et al.* (2025) examine the roles of microRNAs and histone modifications in enhancing stress tolerance in soybean and their applications in molecular breeding.

These articles emphasize how Pangenome analysis can be leveraged to develop crops with higher adaptability to climate change and improved yield potential. Moreover, integrating Pangenome analysis with genome editing and marker-assisted selection can significantly enhance breeding efficiency (Petereit *et al.* 2022). Consequently, these advancements are expected to contribute to the realization of sustainable agriculture and food production that is less affected by environmental fluctuations.

Finally, we would like to express our deepest gratitude to all the researchers who contributed to this special issue and provided valuable insights that will advance this field. We also extend our sincere appreciation to the Editor-in-Chief, Dr. Izawa, for granting us the opportunity to edit this special issue. We hope that this special issue will serve as a foundation for future Pangenome research and its applications in crop breeding.
